# Feline Foamy Virus Infection: Characterization of Experimental Infection and Prevalence of Natural Infection in Domestic Cats with and without Chronic Kidney Disease

**DOI:** 10.3390/v11070662

**Published:** 2019-07-19

**Authors:** Carmen Ledesma-Feliciano, Ryan M. Troyer, Xin Zheng, Craig Miller, Rachel Cianciolo, Matteo Bordicchia, Nicholas Dannemiller, Roderick Gagne, Julia Beatty, Jessica Quimby, Martin Löchelt, Sue VandeWoude

**Affiliations:** 1 Department of Microbiology, Immunology, and Pathology, College of Veterinary Medicine and Biomedical Sciences, Colorado State University, Fort Collins, CO 80523, USA; 2 Division of Infectious Diseases, Department of Medicine, School of Medicine, University of Colorado Anschutz Medical Campus, 12700 E. 19th Ave., Aurora, CO 80045, USA; 3 Department of Microbiology and Immunology, University of Western Ontario, 1151 Richmond St., London, ON N6A 5C1, Canada; 4 Department of Veterinary Pathobiology, Oklahoma State University, Stillwater, OK 74075, USA; 5 Department of Veterinary Biosciences, The Ohio State University, Columbus, OH 43210, USA; 6 Sydney School of Veterinary Science, Faculty of Science, University of Sydney, NSW 2006, Australia; 7 Department of Clinical Sciences, College of Veterinary Medicine and Biomedical Sciences, Colorado State University, Fort Collins, CO 80523, USA; 8 Department of Veterinary Clinical Sciences, The Ohio State University Veterinary Medical Center, 601 Vernon Tharpe Street, Columbus, OH 43210, USA; 9 Department of Viral Transformation Mechanisms, Research Program Infection, Inflammation and Cancer, German Cancer Research Center (DKFZ), Im Neuenheimer Feld 242, 69120 Heidelberg, Germany

**Keywords:** foamy virus, spumavirus, retrovirus, viral tropism, infection, kidney, cats, chronic kidney disease, chronic renal disease

## Abstract

Foamy viruses (FVs) are globally prevalent retroviruses that establish apparently apathogenic lifelong infections. Feline FV (FFV) has been isolated from domestic cats with concurrent diseases, including urinary syndromes. We experimentally infected five cats with FFV to study viral kinetics and tropism, peripheral blood mononuclear cell (PBMC) phenotype, urinary parameters, and histopathology. A persistent infection of primarily lymphoid tropism was detected with no evidence of immunological or hematologic perturbations. One cat with a significant negative correlation between lymphocytes and PBMC proviral load displayed an expanded FFV tissue tropism. Significantly increased blood urea nitrogen and ultrastructural kidney changes were noted in all experimentally infected cats, though chemistry parameters were not outside of normal ranges. Histopathological changes were observed in the brain, large intestine, and other tissues. In order to determine if there is an association of FFV with Chronic Kidney Disease, we additionally screened 125 Australian pet cats with and without CKD for FFV infection and found that FFV is highly prevalent in older cats, particularly in males with CKD, though this difference was not statistically significant compared to controls. Acute FFV infection was clinically silent, and while some measures indicated mild changes, there was no overt association of FFV infection with renal disease.

## 1. Introduction

Feline foamy virus (FFV) is a retrovirus belonging to the ancient *Spumaretrovirinae* subfamily that infects domestic cats (*Felis catus*) and was originally discovered following development of cytopathic effects (CPEs) in feline cell lines [1,2]. Foamy viruses (FVs) cause multiple CPEs in vitro including multinucleation, giant cell formation, and vacuolization, leading to cells looking “foamy” (and where the “*spuma*” originates) [1,3,4,5]. In naturally-occurring and experimental infections of the domestic cat, however, FFV infection does not cause obvious disease, and has not been definitively associated with pathology despite establishing a persistent, life-long infection with a wide tissue tropism [3,6,7,8,9,10,11]. It is believed the apathogenicity of FVs in general is due to long periods of co-evolution with their hosts that has led to a disease-free or highly-attenuated infection [2,12,13]. FV transmission is thought to primarily occur via salivary shedding and ongoing contact between animals, though alternate routes such as vertical transmission through lactating dams have been reported [7,14]. In cats, biting and amicable prolonged contact, such as grooming, have been suggested as routes of transmission [7,15,16]. Global FFV prevalence in pet and feral domestic cats can be high and varies from 8 to 80% based on geographic location, population sampled, and assay type [16,17,18,19,20,21,22,23,24,25,26]. FFV prevalence studies of cats in the USA have documented infection rates of 10 to 75%, with age and male sex identified as risk factors in some cohorts [7,16,27].

FVs are generally host-specific with the exception of simian foamy virus (SFV) where non-human primates may transmit virus to related species and zoonotically to humans [28,29,30,31,32,33,34]. Zoonotic transmission of FFV to humans has not been detected thus far [12,19,35]. Because of the apparent apathogenicity, wide tissue tropism, and large vector cassette packaging capacity, FVs have been used to develop vaccine and gene therapy vectors in multiple species including cats and non-human primates as a model for human therapies [10,13,36,37,38,39,40,41,42]. Many aspects about FV biology, including target cells, latency reservoirs, the specific receptor used for cell entry, viral kinetics over time following infection, and peripheral blood mononuclear cell (PBMC) population changes during infection have been poorly documented [6,12,43,44]. Experimental FFV infection studies in disease-free specific-pathogen-free (SPF) domestic cats with age-matched negative controls using modern and specific assays are rare; the majority have been conducted with domestic cats kept as pets or from shelters [3,6,7,8,11,38,42,45].

While FFV has been detected in apparently disease-free and healthy animals and has historically been considered apathogenic, the virus has been detected in animals suffering from co-infecting pathogens including feline immunodeficiency virus (FIV) [16,17,46,47], feline leukemia virus (FeLV) [45,48,49], feline coronavirus (FCoV) [24,50], and *Bartonella henselae* [18]. German and others reported histopathological changes in kidney and lung following experimental FFV inoculation [6]. A recent study of zoonotic infection with SFV in African hunters found alterations of urinary parameters including blood urea nitrogen (BUN) and serum creatinine, among other hematological changes [30]. These findings in both cats and humans call into question whether chronic infections with FVs are truly apathogenic.

FFV has also been isolated from cats with renal and other urinary tract disease [4,6,24,51,52,53,54,55], polyarthritis [45,47], neoplasia [1,3,24,56], upper respiratory illness [6,24], and myeloproliferative diseases [7]. Chronic kidney disease (CKD) is one of the most commonly diagnosed renal diseases in cats, with prevalence rates reaching up to 85% in geriatric cats [57,58,59]. CKD is characterized by functional and structural loss of kidney tissue likely resulting from prolonged or repeated renal insults [60,61,62,63]. As renal function declines, urine concentrating ability is lost and glomerular filtration rate falls, which eventually manifests as azotemia characterized by elevated BUN and serum creatinine. While the etiologies of CKD are often unknown, a list of comorbidities have been associated with the development of CKD, including retroviral infections [64,65,66,67].

Due to the widespread presence of FFV in domestic cat populations and the knowledge gaps that remain about FFV pathogenicity, especially considering its use in vaccine and gene therapy development, biochemical and histopathologic data from an in vivo FFV experimental infection in healthy SPF domestic cats [10] were further analyzed with emphasis on clinical, immunological, and pathological characteristics and changes during early infection. Due to the renal findings in this study and the detection of FFV in cats suffering from urinary disease, we sought to establish if there is an association between FFV and CKD in cats. We compared FFV prevalence rates in pet cats suffering from CKD in Australia (AU) (as measured by increased blood creatinine) and compared findings to age-matched cats without evidence of CKD. We hypothesized that (1) FFV causes subtle perturbations in immunological and hematological parameters in addition to histopathological changes that could potentially lead to disease in domestic cats, and that (2) FFV is associated with chronic kidney disease in cats. We identified mildly altered hematological and biochemical parameters associated with mild histopathological changes in the lung, brain, and other tissues, and ultrastructural changes in the kidney. We additionally found that while FFV is highly prevalent in AU, there was no direct association between FFV infection and CKD pathology in our sampled population.

## 2. Materials and Methods

### 2.1. Cells and Virus Generation

Plasmid pCF-7 encoding an FFV genome that is replication-competent in vitro and in vivo was used as virus source [10,38]. Crandell feline kidney (CrFK) cells [21,25,68] were used for transfection, viral propagation, and titer determination as described [10]. A CPE end-point dilution assay was used to determine viral titer (50% tissue culture infectious dose, TCID_50_/mL) for cat inoculations [10,69]. CPEs consistent with FFV infection used in our assay include cytomegaly, vacuolization, and syncytia formation [1,3,70].

### 2.2. Animals and Study Design for Experimental FFV Inoculation

Cats were infected with pCF-7-derived FFV as a control group for a previous study testing an experimental molecularly modified chimeric FFV vector [10]. Domestic cats used in our study were acquired from the Colorado State University (CSU, Fort Collins, CO, USA) specific-pathogen-free (SPF) colony, which is free of FFV, and housed in an animal facility at CSU accredited by the Association for Assessment and Accreditation of Laboratory Animal Care International. The trial was approved by the Colorado State University Institutional Animal Care and Use Committee (IACUC) on 5 December 2013 and registered under IACUC protocol #: 13-4104A.

**Figure 1:**
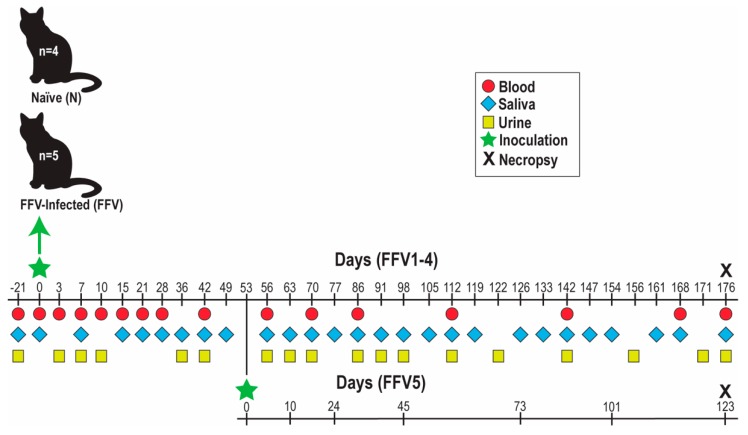
Experimental timeline of feline foamy virus (FFV) (strain pCF-7) inoculation and sample collection in specific-pathogen-free (SPF) domestic cats. Cats were separated into groups based on inoculum type: negative Crandell feline kidney (CrFK) culture media (naïve control cats N1-4) or FFV in CrFK cell culture supernatant (cats FFV1-5). Blood, saliva, urine, and tissues were collected on the dates specified. Sample collection for cat FFV5 was on a different schedule than the rest of the cohort (bottom timeline, adjusted to match rest of FFV cohort). Samples for baseline data were collected on day -21. On day 176 post-inoculation, cats were euthanized to perform necropsy and tissue collection (black **X**). Figure modified with permission from [10].

Nine cats (male castrated and intact females, aged 6–8 months) were separated into naïve (N) and FFV groups based on inoculum (Figure 1, modified with permission from [10]): cats N1-4 received FFV-negative CrFK culture media and cats FFV1-4 were inoculated with 10^5^ FFV particles (derived from a 2.78 × 10^5^ TCID_50_/mL as described previously [10]) in CrFK culture supernatant. Each cat was inoculated with 2 mL, divided into 1 mL intravenously (iv) through the cephalic vein and 1 mL intramuscularly (im) into hindlimb musculature. A fifth cat was inoculated at the start of the study with 10^5^ viral particles (5.56 × 10^5^ TCID_50_/mL) of the afore-mentioned chimeric FFV but remained PCR negative. This cat was subsequently inoculated with 1.4 × 10^6^ TCID_50_/mL of the pCF-7-derived FFV on day 53 of the study and subsequently became FFV PCR positive [10]. This animal, referred to as FFV5, was included in our analyses to increase the statistical power of this study. The study timeline and sample collection schedule (blood, saliva, urine, and tissues) are shown in Figure 1.

Cats were monitored daily for any clinical signs of disease, and rectal temperature and body weight were recorded weekly. Peripheral blood was used for flow cytometric PBMC phenotype analysis or processed to collect serum and plasma shortly after collection. Whole blood and sera were submitted to the CSU Veterinary Diagnostic Laboratory (VDL) for complete blood count (CBC) and chemistry analyses (normal blood urea nitrogen concentration: 18–35 mg/dl; normal serum creatinine concentration: 0.8–2.4 mg/dl). Saliva was collected by swabbing oral mucosa with a sterile cotton-tip applicator and freezing at −80 °C. Urine was collected through cystocentesis and submitted to the CSU VDL for urinalysis, urine sediment, and urine protein:creatinine (UPC) ratio determination. Urine was considered appropriately concentrated if it had a urine specific gravity (USG) over (>) 1.035. UPC ratio was considered normal if below (<) 0.2 and borderline proteinuric if between 0.2 and 0.4 [71]. On day 176 post-inoculation (pi), cats were euthanized and necropsied to assess gross pathology and harvest tissues for virus detection, histopathology, and renal-specific assays at the International Veterinary Renal Pathology Service (IVRPS) in The Ohio State University (OSU, Columbus, OH, USA).

### 2.3. Nested and Real-Time Quantitative PCR for Virus Detection and Quantification

DNA was purified from whole blood, saliva, and plasma using the DNeasy Blood and Tissue Kit (QIAGEN, Hilden, Germany) and amplified using 0.5 µM forward and reverse primers under conditions described [10,72]. Nested PCR (nPCR) products were electrophoresed in agarose gel and stained to identify the desired PCR product. Real-time quantitative PCR (qPCR) was performed in duplicate to triplicate on purified FFV DNA as described [10,72]. Tissue DNA was purified with the DNeasy Blood and Tissue Kit after homogenizing in Buffer ATL and Proteinase K using the FastPrep®-24 Instrument (MP Biomedicals, Santa Ana, CA, USA). Saliva and plasma RNA was purified using the QIAamp Viral RNA Mini Kit (QIAGEN). RNA was reverse transcribed to cDNA using Superscript II and random hexamer primers (Invitrogen, Carlsbad, CA, USA) and resulting cDNA was used for qPCR as described above.

### 2.4. Hematological and Flow Cytometric Analyses for PBMC Phenotyping

Routine hematology and comprehensive PBMC phenotyping were performed at regular intervals (Figure 1). For flow cytometric PBMC phenotype analysis, EDTA-anticoagulated blood was incubated with fluorescent-labelled antibodies (Table S1) diluted at 4 °C flow buffer (PBS with 5% bovine fetal serum and 0.1% sodium azide) and processed by the IMMUNOPREP whole blood lysis method on a Q-Prep EPICS Immunology Workstation (Beckman Coulter, Fort Collins, CO, USA) to lyse red blood cells. Samples were analyzed with a Gallios Flow Cytometer (Beckman Coulter). Output data were analyzed using FlowJo software (FlowJo, Ashland, OR, USA). Data from the CBC were used to determine absolute neutrophil, lymphocyte, and monocyte cell numbers by multiplying the number of nucleated cells by percentages of each cell population. Absolute cell counts were then multiplied by percentages of each cell subpopulation obtained through flow cytometry for each cat per timepoint. PMBC phenotype analyses were divided into two panels: Panel A assayed T lymphocyte populations while Panel B determined number of B cells, natural killer (NK) cells, and monocytes. Markers for activation (CD134+, CD125+, MHCII+) and apoptosis (Fas+) were also assayed. In total, 24 PBMC phenotypes were measured for each cat per timepoint (Table 1). General white blood cell (WBC) activation and apoptosis were determined by multiplying WBC counts by MHCII+ and Fas+ percentages.

### 2.5. Gross Necropsy and Histologic Characterization of Tissues

To evaluate pathologic changes associated with FFV infection, necropsy was performed on cats FFV1-5 and control cat N4 on day 176 pi. The following tissues were collected and stored either frozen at −80 °C for viral tropism determination (qPCR) or in 10% buffered formalin for histopathological evaluation by light microscopy: lymph nodes (submandibular, mesenteric, pre-scapular, retropharyngeal, and ileocecocolic), thyroid, tongue, tonsil, oral mucosa, salivary glands, thymus, heart, lung, spleen, liver, kidney, ovary, testis, mammary tissue, brain (cerebrum, cerebellum, brainstem), small intestine (jejunum, ileum), colon, bone marrow, and hindlimb skeletal muscle. For histopathological assessment, formalin-fixed tissue samples were embedded into paraffin and 5 μm sections were collected onto charged slides (Superfrost; CSU CDL, Fort Collins, CO, USA). One slide of each tissue was stained with hematoxylin and eosin (HE) for microscopic examination. Tissues were scored using the following scale: 0 = no apparent pathology/change, 1 = minimal change (minimally increased numbers of small lymphocytes, plasma cells, macrophages, and/or mast cells), 2 = mild change (mild inflammation, edema, and/or parafollicular expansion, secondary follicle formation, and presence of tingible body macrophages within lymph nodes), 3 = moderate change (as previously described, but more extensive), 4 = marked changes (as previously described, but with severe inflammation, edema, and/or lymphoid reactivity).

### 2.6. Renal Tissue Microscopic and Ultrastructural Examination

Renal tissues collected from cats FFV1-5 and N4 during necropsy were submitted to the IVRPS for comprehensive analysis with light microscopy (LM), transmission electron microscopy (TEM), and immunofluorescence (IF). Samples were submitted in 10% buffered formalin for LM, 3% glutaraldehyde for TEM, and Michel’s transport media for IF, and were processed as previously described [73]. Briefly, formalin-fixed paraffin embedded samples were sectioned at 3 µm thickness and stained with HE, Periodic Acid Schiff (PAS), Masson’s Trichrome (MT), and Jones Methenamine silver method (JMS). Samples for TEM were processed routinely and examined with a JEOL JEM-1400 TEM microscope (JEOL USA, Inc., Peabody, MA, USA) and representative electron micrographs were taken with an Olympus SIS Veleta 2K camera (Olympus Soft Imaging Solutions GmbH, Münster, Germany). For IF, samples were washed to remove residual plasma constituents, embedded in Optimal Cutting Temperature (OCT, Sakura Finetek USA INC, Torrance, CA, USA), and frozen until sectioning. The OCT blocks were sectioned at 5 µm thickness and direct IF performed with FlTC-labeled goat anti-feline Immunoglobulin (Ig) G, IgM, and IgA antibodies (Bethyl Laboratories, Montgomery, TX, USA) as well as FITC-labeled rabbit anti-human lambda light chain (LLC), kappa light chain (KLC), and C1q antibodies (Dako-Agilent, Santa Clara, CA). Stained sections were examined using an Olympus BX51 epifluorescence microscope and representative images were taken with a Nikon Digital Sight DS-U2 camera (Nikon, Tokyo, Japan). TEM assessment was not performed on control cat N4.

### 2.7. Samples from Australian Pet Cats with CKD and Non-Azometic, Age-Matched Controls

To investigate a possible association between FFV infection and naturally-acquired azotemic CKD, biobanked residual samples from pet cats undergoing routine clinical care at the University Veterinary Teaching Hospital Sydney (UVTHS), Sydney School of Veterinary Science, were obtained. Sample collection was approved by the University of Sydney Animal Ethics Committee, approval numbers N00/7-2013/3/6029 (approved 26 August 2013) and 2016/1002 (approved 17 May 2016). Cases were defined as CKD-positive (“CKD”) if serum creatinine was elevated above the reference range, USG (measured concurrently) was <1.035, and clinical signs consistent with CKD were present [71]. Age-matched control samples were from non-azotemic clinically healthy cats with USG > 1.050.

A total of 125 samples (53 whole blood and 34 plasma samples in the CKD group and 38 whole blood samples in the control group) were analyzed for FFV infection by either nPCR (whole blood) or specific FFV ELISA against Gag antigen (plasma) as described previously [10]. Detection of FFV antibodies in sera/plasma is more sensitive for detection than FFV PCR [8,10,16,26,72]. Four different laboratories were used for serum creatinine and BUN determination and values were classified as abnormal based upon the references established for each laboratory: BUN: 7.2–10.7 mmol/L (20.16–29.96 mg/dl), 5.7–12.9 mmol/L (15.96–36.12 mg/dl), 5–15 mmol/L (14–42.01 mg/dl), or 3–10 mmol/L (8.4–28 mg/dl) [74]; serum creatinine: 91–180 µmol/L (1.03–2.04 mg/dl), 71–212 µmol/L (0.8–2.4 mg/dl), 40–190 µmol/L (0.45–2.15 mg/dl), or 80–200 µmol/L (0.9–2.26 mg/dl) [75].

### 2.8. Statistical Analyses

For the experimentally FFV-inoculated cats, two-tailed Student’s t test were performed on hematology, flow cytometry, BUN, serum creatinine, and USG data sets. *P*-values < 0.05 were considered statistically significant. For cat FFV5, the timeline following re-inoculation on day 53 with FFV was adjusted so that day 53 equaled day 0 pi. To have consistent timelines with the main FFV group, timepoints after day 53 for cat FFV5 were grouped with either the equivalent day or the nearest date post-inoculation to the cats in the FFV cohort. A Pearson’s correlation coefficient was calculated to determine presence of a correlation and its significance between lymphocyte population numbers and FFV proviral load over time. To assess distributions of viral load to lymphocyte counts, we ran a generalized linear mixed model (GLMM) with the individual cat as a random factor and lymphocyte count as a fixed factor. Data were only run through the GLMM if viral load was at detectable levels and the data fit a negative binomial distribution.

For the CKD analyses, risk ratios and chi-square tests were performed to assess the independence of three pairs of categorical variables: (1) sex and FFV infection, (2) sex and CKD, and (3) FFV infection and CKD. For each pair of variables, cats were stratified by sex (M or F), FFV infection status, and CKD status. Given that both nPCR and ELISA were used to determine FFV infection, CKD analyses were performed on both assays’ results to see if conclusions differed. If a chi-square test produced a *P*-value < 0.05, risk ratios and 95% confidence intervals (95% CI) were calculated as an additional post-hoc test. Risk ratios describe the probability of a health outcome occurring in an exposed group to the probability of the event occurring in a comparison, non-exposed group. A Risk ratio >1 suggests an increased risk of that outcome in the exposed group, and a risk ratio <1 suggests a reduced risk in the exposed group. Cats for which sex data were not known (*n* = 5) were omitted in sex-specific data analyses.

Excel (Microsoft Corporation, Redmond, WA, USA) and Prism (GraphPad, La Jolla, CA, USA) were used to conduct the Student’s t tests, calculate the lymphocyte and proviral load Pearson’s correlation coefficient, and produce graphs. GLMM and CKD analyses were run using the statistical program R version 3.4.2 [76]. The “fitdistrplus” package [77] was used to determine error distributions of the viral load data and the “glmmTMB” package [78] was used to run the GLMM. Chi-square tests and RRs for CKD were calculated using the ‘epitools’ package [79].

## 3. Results

### 3.1. FFV-Infected Cats Did Not Show Clinical Signs of Infection Despite a Persistent FFV Proviral Load and Specific Humoral Response

**Figure 2:**
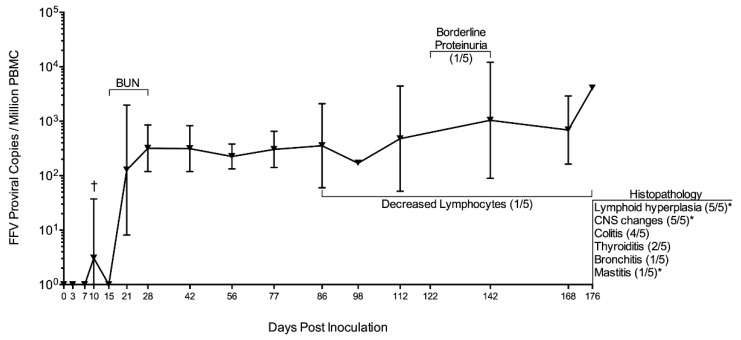
FFV proviral load in PBMC of cats FFV1-5 with summary of significant findings. FFV-infected cats began showing PBMC provirus 21 days pi. Cat FFV5 was re-inoculated and its timeline adjusted to match the rest of the cohort; this cat showed FFV positivity on day 10 post-reinoculation (†) [10]. Blood urea nitrogen (BUN) was significantly increased in infected cats compared to naïve on days 15, 21, and 28. Cat FFV3 had decreased lymphocytes compared to the rest of its cohort, which was negatively correlated to proviral PBMC load (Results Section 3.3). FFV3 also had borderline proteinuria on days 122 and 142 pi. Histopathological changes found after necropsy on day 176 are shown at the right-hand margin. Graph shows mean of FFV group cats’ FFV proviral load with bars denoting standard deviation. Numbers in parenthesis indicate number of cats out of the FFV cohort showing findings, with an asterisk (*) indicating findings also observed in control cat N4 to a lesser severity.

As previously reported, all FFV group cats became PBMC FFV DNA positive (PCR), starting at 21 d pi (Figure 2) [10]. One cat (FFV3) was not PCR positive until day 42 but maintained a much higher proviral load than the rest of the cohort from that point on (Figure 5 in [10]). Cat FFV5 was FFV PCR positive by 10 days pi with FFV pCF-7 (Figure 2). FFV DNA was consistently detected in PBMC once the animals showed positivity [10]. Out of 80 FFV saliva samples tested, FFV RNA was detected only once (cat FFV4 on day 133 pi, Table 2); this sample was FFV DNA negative. All plasma samples tested were negative for FFV RNA and DNA (Table 2). Cats in the naïve group remained negative at all times. Additional proviral kinetics and anti-FFV antibody responses were reported previously [10].

Despite evidence of productive infection and specific immune response [10], none of the cats developed a fever, had changes in body weight, or displayed signs of clinical illness related to infection (such as anorexia or lethargy). CBC and chemistry values did not change significantly from the pre-inoculation time point (day -21) or indicate disease [80].

### 3.2. FFV Provirus Tissue Tropism Is Primarily Lymphoid

FFV DNA was detected in the tissues of four out of the five FFV-inoculated cats primarily in lymphoid tissue including lymph nodes (submandibular, retropharyngeal, and prescapular, which drain lymph from head, neck, and forelimbs), tonsil, and spleen (Table 3). Cat FFV3 showed an expanded tissue tropism to central lymphoid tissues (thymus and bone marrow) in addition to non-lymphoid tissue (oral mucosa). The prescapular lymph node was the tissue with highest viral load (cat FFV1). Cat FFV5 showed an FFV tissue tropism similar to the rest of the FFV group, with the submandibular lymph node having the highest viral load (Table 3). Control cat N4 and cat FFV4 did not have detectable provirus in any of the tissues tested.

### 3.3. Significant PBMC Phenotypic Changes Were Rare though a Negative Correlation Was Found between Lymphocytes and FFV Proviral Load in Cat FFV3

Out of the 24 cell populations and activation or apoptosis markers assayed for each cat per timepoint (Table 1), there were only 9 instances where significant differences (*P* < 0.05) were found between infected and control animals (Table 2). Significantly increased populations were found between FFV (1–5) and N (1–4) groups in the following instances: (1) absolute monocyte numbers on days 15 (*P* = 0.036) and 42 (*P* = 0.025) pi and (2) CD21+MHCII+ cells on d 86 pi (*P* = 0.0076). FFV-group cats had decreased populations compared to controls in the following instances: (1) CD8+CD25+ cells on d 112 pi (*P* = 0.044), (2) CD8+CD134+ cells on d 10 pi (*P* = 0.031), (3) CD8+FAS+ on d 10 pi (*P* = 0.015), (4) CD56+ cells on d 112 pi (*P* = 0.00038), and (5) CD56+MHCII+ cells on days 15 (*P* = 0.049) and 112 pi (*P* = 0.00070).

**Figure 3:**
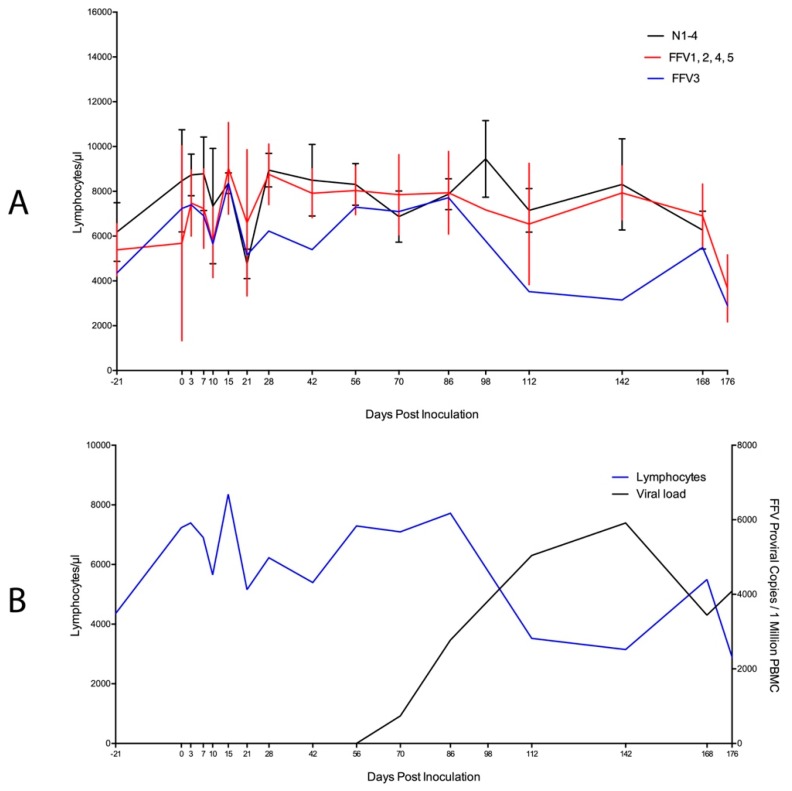
High viral load correlates with decline in circulating lymphocytes in cat FFV3. (**A**) Absolute lymphocyte population numbers determined through complete blood count for cat FFV3 (blue line) appeared to decrease over time compared to all other cats in the study. Naïve cats are grouped on the black line and the rest of the FFV-group cats are displayed on the red line; (**B**) A significant negative correlation (*r* = −0.653, *P* = 0.006) was found between lymphocytes and FFV proviral load [10] in cat FFV3 as lymphocyte population numbers (blue line) decreased and proviral load (determined by qPCR, black line) increased over time. Bars denote standard deviation.

We further evaluated WBC populations in cat FFV3 due to the altered PBMC FFV provirus pattern observed [10]. This cat appeared to have lower lymphocytes and a trend for decreasing lymphocyte count over time compared to the rest of the infected and naïve cats (Figure 3A, blue line) as PBMC proviral load increased over time (Figure 3B, black line). A Pearson’s correlation coefficient test for this cat showed a significant negative correlation between lymphocyte cell number and viral load over time (*r* = −0.653, *P* = 0.006). There was no correlation found in the rest of the infected cats (data not shown) and there was no significant relationship between viral load and lymphocyte count when we analyzed all cats as a group (GLMM Estimate −0.530, *P* = 0.596).

### 3.4. Subtle Differences in Urine and Hematological Parameters Were Detected between Experimentally Infected and Control Cats

**Figure 4:**
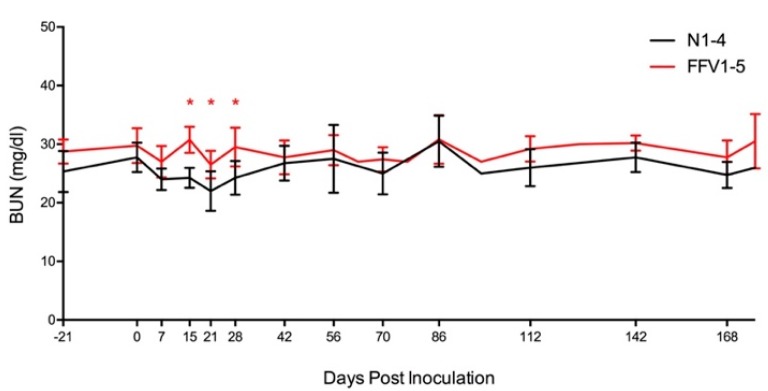
Blood urea nitrogen (BUN) levels are within normal range, but higher in FFV-infected versus naïve control cats. While BUN, one of the biomarkers used to assess renal health, remained within normal range (18–35 mg/dl) for all cats, concentrations tended to be higher in infected cats (red line) compared to naïve cats (black line) on days 15, 21, and 28 pi (red asterisks, *P* < 0.05). Lines represent mean of BUN measurements for the cats in each group. Vertical lines denote the standard deviation for each grouped measurement.

UPC ratios were 0.1 (normal) for all cats throughout the study, with the exception of two timepoints in cat FFV3 (d 122 and 142 pi) where its UPC ratio increased from 0.1 to 0.2 (borderline proteinuric), before decreasing back to normal (0.1) on day 176 pi (Figure 2) [81]. This mild transient increase in UPC coincided with the timepoint when this cat’s PBMC FFV proviral load was highest (d 142 pi) (Figure 3B) [10]. BUN concentration remained within normal ranges (18–35 mg/dl) for all cats throughout the study, however values were higher in infected cats compared to naïve controls on three consecutive timepoints: days 15 (*P* = 0.012), 21 (*P* = 0.039), and 28 (*P* = 0.025) (Figure 3 and Figure 4). All cats had USG > 1.035 and urinalyses and urine sediment were unremarkable throughout the study. Serum creatinine concentrations were at or below 1.8 mg/dl at all timepoints, and there were no significant differences in serum creatinine between infected and control cats [82].

### 3.5. Mild Lymphoplasmacytic Infiltrates and Lymphoid Hyperplasia of Multiple Tissues Were Associated with FFV Exposure

**Figure 5:**
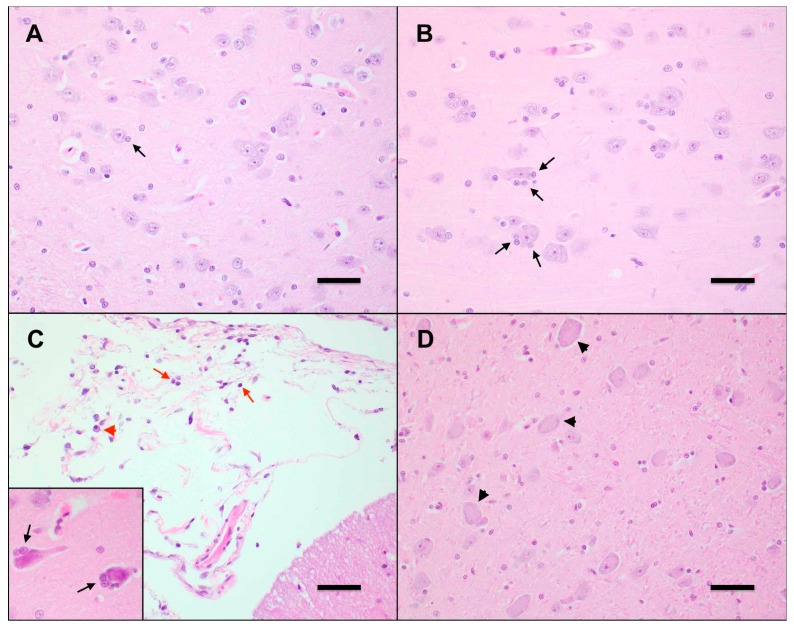
FFV-infected cats exhibit early neurodegenerative changes in the central nervous system. (**A**) Neurons in the CNS of control cat N4 contain uniform, round nuclei, abundant basophilic Nissl substance, and are flanked by few glial cells (black arrow). Frontal lobe, Hematoxylin-eosin (HE) 400×. Scale bar = 100 µm. (**B**) Neurons in the CNS of FFV-infected cat FFV3 exhibit moderate satellitosis, characterized by increased numbers of glial cells (black arrows). Thalamus, HE 400×. Scale bar = 100 µm. (**C**) The meninges of FFV-infected cat FFV5 are expanded by minimal numbers of mature small lymphocytes (red arrows) and plasma cells (red arrowheads). Cerebellum, HE 400×. Scale bar = 100 μm. Neurons in the frontal lobe of this animal (inset) are shrunken, with hypereosinophilic cytoplasm, and exhibit moderate satellitosis (black arrows). Frontal lobe, HE 400×. (**D**) Neurons in the CNS of an FFV-infected cat are swollen and rounded, with an indistinct nucleus and a dispersed Nissl substance (chromatolysis). Thalamus, HE 400×. Scale bar = 100 µm.

No significant clinical or pathological findings were grossly observed in control (N4) or FFV-infected cats (FFV1–5) during necropsy. Microscopic evaluation of tissues from FFV-infected cats revealed mild (*n* = 3) to moderate (*n* = 2) lymphoid hyperplasia in retropharyngeal, submandibular, mesenteric, and prescapular lymph nodes, characterized by numerous secondary follicles that contain abundant tingible-body macrophages. The tonsils of infected animals exhibited minimal (*n* = 1), mild (*n* = 3), and moderate (*n* = 1) lymphoid hyperplasia with multifocal infiltration of small numbers of lymphocytes beyond the capsule in one mildly affected animal. Two infected cats exhibited mild thyroiditis characterized by multifocal infiltrates of small lymphocytes, plasma cells, and macrophages within the interstitium and surrounding colloid-filled follicles of varying size. Within the ileum, Peyer’s patches were minimally (*n* = 1) to mildly (*n* = 4) hyperplastic, and one animal exhibited multifocal lymphoplasmacytic infiltrates extending deep into the submucosa. Additionally, minimal (*n* = 3) to mild (*n* = 1) lymphoplasmacytic colitis was observed in FFV-infected cats, with lymphoplasmacytic infiltration into the submucosa that caused disruption of the submucosal architecture (*n* = 3), as well as small numbers of degenerate neutrophils scattered within the submucosa (*n* = 1). In the cerebrum of FFV-infected cats, there were minimally (*n* = 2) to mildly (*n* = 3) increased numbers of glial cells (gliosis) and paired astrocytes (astrocytosis) surrounding scattered neurons within the gray matter (satellitosis), a feature that was most prominently noted within the frontal lobe and thalamus (Figure 5B). Cat FFV5 also had small numbers of small lymphocytes within the meninges (lymphocytic meningitis) (Figure 5C). Scattered neurons within these regions were multifocally swollen, rounded, and demonstrated mild central dispersion of Nissl substance (chromatolysis), as well as rare, scattered neurons that exhibited hypereosinophilic and/or fragmented cytoplasm (potentially indicative of neuronal necrosis) (*n* = 2) (Figure 5D). One cat had mild multifocal lymphohistiocytic mastitis. Histologic changes in cat FFV5 were slightly more pronounced when compared to the other infected animals and included moderate lymphoid hyperplasia in the tonsil with moderate numbers of lymphocytes and macrophages within the tonsil medullary sinus, and enlarged germinal centers in the Peyer’s patches. Within the lung of this cat, the parabronchial interstitium and alveolar septa were multifocally expanded by small numbers of small lymphocytes, intact neutrophils, and macrophages (interpreted as mild interstitial pneumonia). Alveoli were occasionally filled with small numbers of alveolar macrophages and frequently lined by plump, cuboidal epithelial cells, indicating type 2 pneumocyte hyperplasia, with occasional clubbing of alveolar walls due to mild smooth muscle hypertrophy.

Non-specific histologic findings in control cat N4 included mild lymphoid hyperplasia in the mesenteric lymph node, tonsil, and thymus, minimal to mild inflammatory infiltrate in the tongue, and mammary tissue, and minimal chromatolysis in the cerebrum. Findings in this control cat ranged from very subtle to mild, and were less severe than in infected animals.

### 3.6. Ultrastructural Changes Were Noted in the Kidneys of FFV-Infected Cats

Histopathology of the kidneys from cats FFV1-5 (Table 4) demonstrated a few small foci of tubular degeneration encompassing fewer than 15 tubular cross-sections per focus in cats FFV1 and FFV2; cat FFV1 also had associated atrophy of the tubules. Glomeruli from the remaining cats in the FFV cohort and control cat N4 were within normal limits.

**Figure 6:**
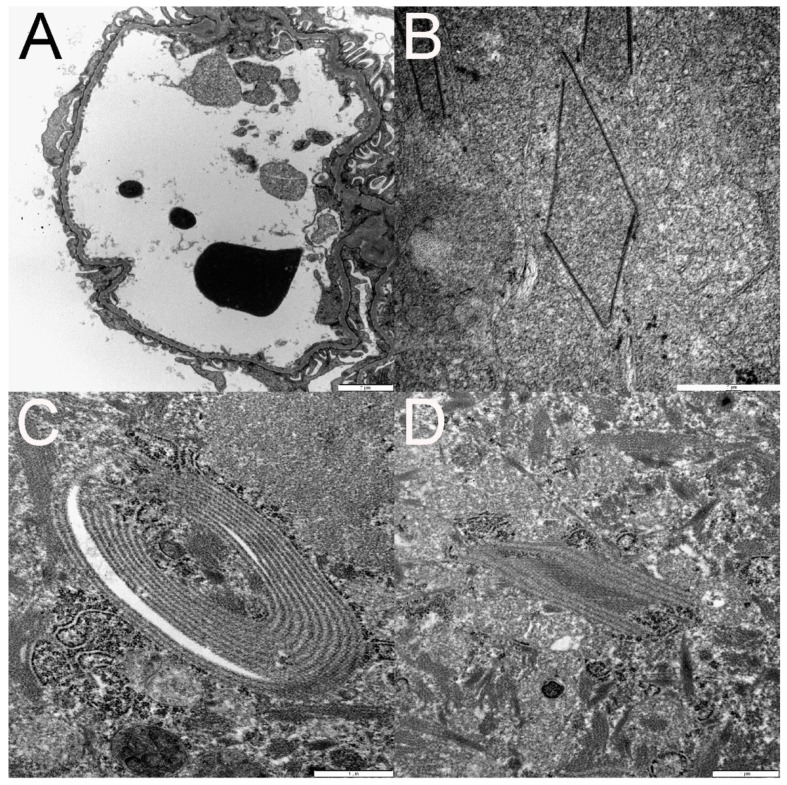
Transmission electron microscopy (TEM) documents podocyte foot process effacement (**A**). Examples of organized linear structures in tubular epithelial cell cytoplasm are depicted in panels **B**–**D**. These structures ranged from polygonal (**B** and **D**) to ovoid (**C**). Some structures were composed of a single electron dense line (**B**), whereas others were composed of numerous parallel electron dense lines (**C**,**D**) separated by regularly spaced electron lucent lines (**C**,**D**).

Ultra-structural TEM evaluation of glomeruli from cats FFV1-5 demonstrated minimal to moderate segmental effacement of podocyte foot processes in all infected cats (Figure 6A and Table 4). There were a few small segments of wrinkled glomerular capillary walls in cat FFV5 (Table 4). Electron-dense whorls resembling myelin figures appeared free in the cytoplasm or within cytoplasmic vacuoles in tubular epithelial cells of three of the infected cats (Table 4). Cytoplasmic vacuolization of parietal or tubular epithelial cells was present in two cats (Table 4).

Within the cytoplasm of the proximal tubular epithelial cells of four of these cats, there were small electron-dense spirals and linear structures of 10–15 nm in length arranged in pairs, stacks, polygonal shapes, or spirals, and of variable length (Figure 6B–D). Sometimes the linearly shaped ones had a beaded appearance or formed structures resembling a zipper. Mitochondria occasionally wrapped around the structures. In cat FFV5, the structures were similar to the ones found in the other FFV cats but appeared significantly more organized. TEM evaluation was not conducted on cat N4.

Immunofluorescence did not demonstrate definitively positive (granular) labeling for any of the antibodies (IgG, IgM, IgA, LLC, KLC, and C1q). Cat FFV3 had weak blush to linear staining with IgM of the glomerular mesangium and some capillary walls but based on the pattern of staining, it was considered non-specific. Immunofluorescence was negative in tissues from control cat N4.

### 3.7. FFV Is Highly Prevalent in Australian Domestic Pet Cats

**Figure 7:**
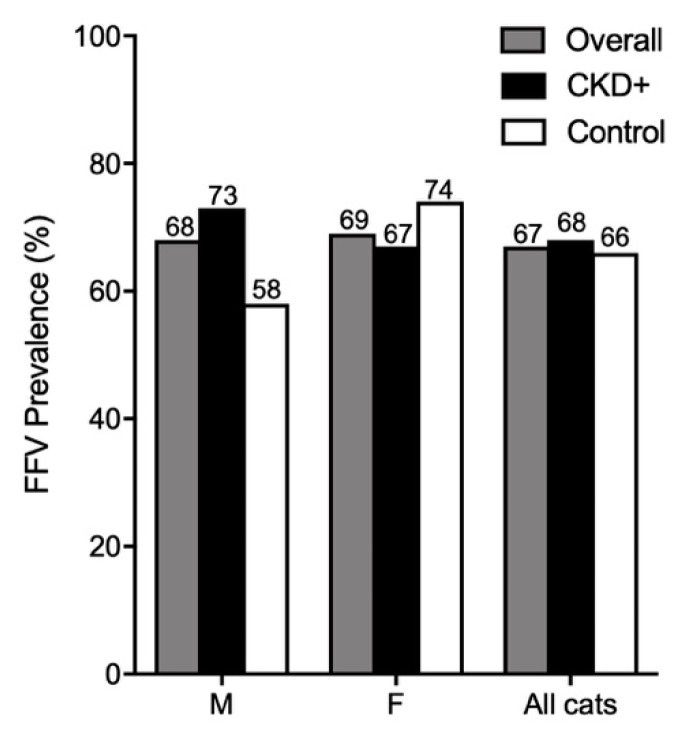
FFV prevalence is high in Australian domestic pet cats. Males with CKD had higher FFV prevalence than male control cats, although not significantly. M = male; F = female.

We determined FFV prevalence by either nPCR or ELISA in groups of cats with and without CKD to evaluate associations between FFV infection and the incidence of CKD. Overall FFV prevalence was 67% (Table S2) with no significant difference between CKD and control groups (Figure 7). Overall FFV prevalence was similar between males (68%) and females (69%). Males had a higher FFV prevalence in CKD (73%) versus males without CKD (58%), although this difference was not significant (Figure 7).

Chi-square test statistics and P-values are reported in Table S2. Our results suggest there is no significant association between CKD and FFV infection. Chi-square and relative risk ratio results did not differ between nPCR and ELISA indicating that neither assay detected an association between CKD and FFV infection in all groups evaluated.

## 4. Discussion

This study further characterized early FFV infection and host immune response in healthy SPF domestic cats following experimental FFV inoculation using a well-characterized replicative FFV molecular clone that achieves comparable viral titers, similar gene expression and comparable humoral immune response to wild-type virus in experimentally inoculated cats [42]. Though it is difficult to exactly recapitulate natural exposure route and dose, the FFV experimental exposure protocol used in this study is comparable to inoculation doses others have used in experimental FFV inoculation studies, and resulted in viral loads and antibody responses comparable to those recorded in naturally infected animals [6,8,11,42]. In addition to clinical monitoring, assessing viral kinetics and tropism, determining specific antibody response, and a histopathological assessment of different tissues, we conducted assays to expand infection characterization. This included flow cytometric assessment of specific white blood cell subsets suggested to be involved in FFV infection and renal-specific assays to determine the extent of FFV involvement in renal health or disease. Samples analyzed were obtained from control experimental cats reported in a previous study in which an FFV-based vaccine candidate was tested [10]. Based on microscopic findings from the FFV-infected cohort showing evidence of injury in renal tissues during acute FFV infection (Figure 6A and Table 4), we investigated the potential association of FFV with CKD in Australian client-owned cats following natural FFV infection.

FFV established a detectable, persistent, and clinically apathogenic infection during the relatively acute 6-month time period of our study [6,8,10,38]. Provirus was primarily isolated from lymphoid tissues including PBMC, the retropharyngeal lymph node, and spleen as previously reported (Table 3) [6,11,27]. The expanded tissue tropism found in cat FFV3, with 10-fold higher PBMC proviral load than the other cats, also included the oral mucosa, suggesting that animals with higher viral loads could have dissemination to non-lymphoid sites. We were unable to detect virus in the tissues of one cat (FFV4) which also tended to have lower PBMC proviral load [10]. Viral RNA was detected once in the saliva of cat FFV4 (Table 2), indicating that limited amounts of virus was being shed, and that salivary excretion may not correlate with widespread tissue distribution. A recently published report by Cavalcante and others on FFV-infected cats in Brazil found that pet and feral cats were more likely to be FFV positive in PBMC than in buccal swabs, as tested by PCR [83]. This has also been reported in primates due to a delay in salivary excretion of virus compared to blood [84]. A wide variability of FV positivity has been shown in different nonhuman primate species and even within the same species [85,86,87]. It is possible that due to the acute time period in our study, viral titer had not yet reached high enough levels in the oral cavity to be detected in the saliva.

A significant negative correlation between lymphocyte numbers and proviral load was found in cat FFV3 (Figure 3B), despite the fact that PBMC phenotype analysis did not indicate increased cell death or lymphocyte population contraction in FFV-infected cats (Table 2). Thus, PBMC phenotyping appears not to be a useful indicator of FFV infection. These findings, however, indicate that a subset of FFV-infected cats may experience higher viral loads with the potential for concurrent lymphocyte decline. Further work analyzing correlates of FFV-infected cats with hematologic indices is warranted to investigate this limited observation.

Necropsy and histologic analysis of experimentally infected cats yielded minimal to moderate changes in the lymphoid compartment, CNS, large intestine, lung, and thyroid. FFV-associated lung lesions have previously been noted in another experimental FFV infection study which reported mixed cellular infiltrates and eosinophilic fluid within alveolar walls [6], similar to findings reported here. Alterations in CNS histopathology of FFV-infected cats suggest viral replication or associated inflammation as is seen in other retroviral infections [88,89,90]. FFV has previously been isolated from the CNS of cats, and may therefore indicate that FFV is capable of productive CNS infection and subtle neurologic alterations [91]. These findings suggest a potential role for FFV in the development of mild acute inflammation in a variety of parenchymal organs and brain. FFV proviral load did not appear to correlate to the presence or severity of histopathology and histopathological changes were observed in tissues that were not FFV PCR positive and vice-versa. The changes observed were mild and consistent with inflammation due to nonspecific immune stimulation, and cannot be unequivocally associated with FFV infection, which would be ideally confirmed by viral propagation from isolated tissues or immunohistochemistry [11]. Nevertheless, the findings of consistent mild inflammation in multiple tissues of SPF cats exposed to FFV suggest that microscopic alterations occur during acute FFV infection.

FFV has previously been associated with urinary syndrome pathology [4,6,24,51,52,53]. BUN concentrations remained normal during FFV-infection, but were statistically elevated in FFV-infected compared to naïve groups on days 15, 21, and 28 pi, which coincided with the days when animals were first FFV PCR and ELISA positive [10]. These findings are intriguing given recent reports of chronic zoonotically SFV-infected people with increased BUN compared to un-infected controls [30]. Borderline proteinuria (increased UPC ratio to 0.2 [71]) was also recorded in cat FFV3 on days 122 and 142 pi, which coincided with the timing of the highest viral load measured in the study. While recorded changes in BUN and UPC ratio are interesting, these findings may not specifically indicate renal proteinuria and could be considered a transient systemic response to infection.

Ultrastructural kidney changes (glomerular podocyte foot process effacement, myelin figures, vacuolization, and wrinkled glomerular capillary walls, Figure 6A and Table 4) are non-specific and reversible changes. If enough podocytes are irreversibly injured, then the patient can develop segmental to global glomerulosclerosis, a disease process in humans and small animals that can cause proteinuria, usually with UPC >2. Although a cut-off for the number of irreversibly injured podocytes has not been established in cats, a model of glomerulosclerosis in rats estimated that >40% of the podocytes have to die and detach in order for glomerulosclerosis to develop [92]. Notably, glomerulosclerosis was not identified in any of the cats in the present study. Tubular atrophy noted on histopathology is an irreversible lesion and often seen in cats with clinical evidence of CKD [62,63]. Overall, our findings indicate subtle reversible renal alterations, indicating that FFV does not induce dramatic changes to renal function within 6 months of infection, however it is unknown how the lesions could have progressed in a chronic infection. We did not detect correlations between proviral PBMC load and presence or severity of TEM changes.

Electron-dense structures identified in proximal tubular epithelial cells in the kidney (Figure 6B–D) could represent viral structures at immature stages of assembly before forming the spherical shapes of FFV virions reported in the literature [1,93,94]. Similar structures have been found in the central nervous system in both cats and humans. Cook and others described similar tubular structures as “paramyxovirus nucleocapsid-like” in the cytoplasm of oligodendrocytes taken from demyelinating lesions in the optic nerve of three clinically healthy adult cats [95]. These inclusion-body like structures were of 16–17 nm in diameter and fused into penta- to septa-laminar shapes and 900 nm in length. The authors suggested a possible viral etiology. Wilcox and others (1984) reported similar structures in optic nerves and brains of 24 clinically healthy cats from which FFV was isolated [91]. While also finding 10–18 nm wide and 500 nm long structures, they also reported structures in much smaller shapes, appearing as “short, disorganized fragments,” located next to where budding virions were observed, in addition to intranuclearly. These structures were, however, found in the cytoplasm of cells that did not display CPEs and thus these lesions were not attributed to FFV but perhaps a morbillivirus [91].

FFV was highly prevalent in Australian domestic cats as reported previously [16,19], and could be due to the lifestyle of Australian cats which are commonly allowed outside [96,97], allowing more opportunities to interact with other potentially infected cats. The samples analyzed also consisted of sera/plasma assayed by ELISA and whole blood analyzed by PCR. PCR is not as sensitive as ELISA for detection and can yield false negatives [8,10,16,26,72], therefore it is possible that FFV prevalence is even higher in Australia. Similar prevalence rates in males (68%) and females (69%) indicates that there are no sex-related differences in prevalence rates in Australian pet cats [16]. A recent study of feral cats in the USA found an association between FFV infection and male sex [27]. However, our epidemiological results were obtained from client-owned desexed animals. Another epidemiological study of FFV in Australia did not find an association between sex and FFV infection in desexed domestic cats, but did see higher incidences of FFV in female feral cats [16]. Our findings suggest that while overall there appears to be no association between FFV and CKD, CKD in male cats may be associated with FFV infection, though any effect is mild, and evaluation of a much larger cohort is required to assess actual associations. Male sex has not been found to be an overall risk factor for CKD, however males can be overrepresented in certain age groups [98].

## 5. Conclusions

Collectively, our findings reinforce and expand on the current established notion that FFV is widely prevalent and apathogenic over an acute time period, supporting decades long assumptions that FFV is well adapted to the domestic cat host. Evaluation of a multitude of hematologic and immunological parameters did not reveal significant host responses to infection. However, our detailed analysis of serum chemical and histopathological changes indicates sub-clinical alterations that could contribute to metabolic or degenerative diseases over time, supporting our hypothesis and work conducted by earlier researchers, and recent reports in humans [1,3,4,6,7,24,30,45,47,51,52,53,54,55,56]. The negative correlation between lymphocyte count and viral load in one cat with higher viral load suggests that a differential susceptibility and potential pathogenicity may exist in some individuals. Studies with larger cohorts of animals with well-characterized disease phenotypes may reveal subtle relationships between FFV and feline health, since the virus is clearly widely distributed in free-ranging cat populations.

## Figures and Tables

**Table 1:** White blood cell (WBC) populations assayed for peripheral blood mononuclear cell (PBMC) phenotype analysis.

Assay	Population	Cluster of Differentiation
CBC	WBC	-
	Monocytes	-
	Lymphocytes	-
	Neutrophils	-
Flow Cytometry (Panel A)	Th ^1^ lymphocytes	CD4+
	Activation	CD4+CD25+
	Activation	CD4+CD134+
	Apoptosis	CD4+Fas+
	Tc ^2^ lymphocytes	CD8+
	Activation	CD8+CD25+
	Activation	CD8+CD134+
	Apoptosis	CD8+Fas+
	Double-positive T cells	CD4+CD8+
Flow Cytometry (Panel B)	B cells	CD21+
	Activation	CD21+MHCII+
	Apoptosis	CD21+Fas+
	NK cells	CD56+
	Activation	CD56+MHCII+
	Apoptosis	CD56+Fas+
	Monocytes	CD14+
	Activation	CD14+MHCII+
	Apoptosis	CD14+Fas+
	WBC activation	MHCII+
	WBC apoptosis	Fas+

^1^ T helper, ^2^ T cytotoxic.

**Table 2:** Summary of findings for diagnostic assays used in the experimental inoculation experiments. Bold font indicates that at least one cat was positive for the measured value, or differences in values between naïve and FFV-infected animals were significant. Cat FFV5 was on a different inoculation and sample collection schedule following re-inoculation on day 53 pi (Figure 1).

Assay	Days Tested	Summary of Findings
Saliva qPCR (RNA)	36, 42, 49, 56, 63, 86, 112, 119, 126, **133**, 142, 147, 154, 161, 168, 176	D133 saliva sample was FFV DNA-negative.
Plasma qPCR (DNA)	42, 86, 142, 176	FFV not detected.
Plasma qPCR (RNA)	15, 56, 112	FFV not detected.
Tissue qPCR (DNA)	176	Virus shows primarily lymphoid tropism. Cat FFV3 had expanded tropism to other lymphoid and non-lymphoid tissues (Table 3). FFV not detected in cat FFV4’s tissues.
CBC, Chemistry	−21, 0, 3, 7, 10, 15, 21, 28, 42, 56, 63, 70, 77, 86, 98, 112, 126, 142, 154, 168, 176	Not indicative of disease.
PBMC Phenotype	−21, 0, 3, 7, **10**, **15**, 21, 28, **42**, 56, 70, **86**, **112**, 142, 168	Significantly increased populations in FFV cats included monocytes and CD21+MHCII+, while CD8+CD25+, CD8+CD134+, CD8+FAS+, CD56+, and CD56+MHCII+ cells were decreased.
BUN, Creatinine	−21, 0, 7, **15**, **21**, **28**, 42, 56, 63, 70, 77, 86, 98, 112, 126, 142, 154, 168, 176	While BUN remained within normal limits for all cats, values were significantly increased in FFV group cats compared to naïve on bolded days. Creatinine values were within normal ranges and did not rise above 1.8 mg/dl.
Urinalysis	−21, 3, 7, 10, 42, 56, 63, 70, 86, 91, 98, 112, 122, 142, 156, 171, 176	USG > 1.035 for all cats. Urinalysis and urine sediment were unremarkable.
UPC Ratio	36, 70, 86, 91, 98, **122**, **142**, 156, 171, 176	UPC ratio was 0.1 (normal) for all cats, except cat FFV3 where it increased to 0.2 (borderline proteinuric) on 122 and 142 days pi, during highest PBMC proviral load for this cat [10].

**Table 3:** FFV provirus has a primarily lymphoid tissue tropism. Viral load was determined through DNA qPCR and is presented as viral copies per million cells. Cat FFV3 had altered PBMC FFV DNA kinetics and expanded tissue tropism compared to the other FFV cats. Cat FFV5 was on a different inoculation schedule than the rest of the FFV cats (Materials and Methods Section 2.2 and Figure 1). Bold text indicates difference in either proviral load or viral detection compared to other cats in the group.

Tissue	N4	FFV1	FFV2	FFV3	FFV4	FFV5	Total Cats
Salivary gland	-	-	-	-	-	-	0
Tongue	-	-	-	-	-	-	0
Oral Mucosa	-	-	-	**2.10 × 10^2^**	-	-	1
Tonsil	-	2.41 × 10^2^	4.03 × 10^2^	-	-	5.10 × 10^2^	3
Prescapular LN	-	**5.89 × 10^3^**	-	-	-	4.96 × 10^2^	2
Submandibular LN	-	3.35 × 10^2^	2.26 × 10^2^	-	-	5.78 × 10^2^	3
Retropharyngeal LN	-	1.86 × 10^2^	1.19 × 10^2^	1.49 × 10^2^	-	2.35 × 10^2^	4
Mesenteric LN	-	-	-	-	-	-	0
Thymus	-	-	-	**3.48 × 10^2^**	-	-	1
Spleen	-	5.93 × 10^2^	3.11 × 10^2^	2.10 × 10^2^	-	3.35 × 10^2^	4
Ileum	-	-	-	-	-	-	0
Bone marrow	-	-	-	**6.10 × 10^2^**	-	-	1
Kidney	-	-	-	-	-	-	0
Muscle	-	-	-	-	-	-	0

**Table 4:** Summary of pathological findings in glomeruli and tubulointerstitial compartment of control and FFV-infected cats. Kidney tissue was collected during necropsy on day 176 and submitted to the International Veterinary Renal Pathology Service for analysis. Cat FFV5 was on a different inoculation schedule than the rest of the FFV cats (Materials and Methods Section 2.2 and Figure 1).

Analysis	Finding	N4	FFV1	FFV2	FFV3	FFV4	FFV5	Total Cats
Histology	Tubular degeneration (+/− atrophy)	-	+	+	-	-	-	2
TEM	Podocyte effacement	NT	+/+	++	++	+	+++	5
	Cytoplasmic electron dense figures	NT	-	+	+	+	+	4
	Cytoplasmic myelin figures	NT	+	+	-	+	-	3
	Cytoplasmic vacuolization	NT	-	+	+	-	-	2
	Wrinkled glomerular capillary walls	NT	-	-	-	-	+	1

+ = minimal, +/+ = minimal to mild, ++ = mild, +++ = moderate, NT = not tested.

## Supplementary Materials

The following are available online at https://www.mdpi.com/1999-4915/11/7/662/s1, Table S1: Antibody marker combinations used for PBMC phenotype analysis through flow cytometry. Table S2: FFV prevalence and chi-square analysis data for CKD studies.
