# Supplementary material for: Feline Foamy Virus Infection: Characterization of Experimental Infection and Prevalence of Natural Infection in Domestic Cats with and without Chronic Kidney Disease
Source: Viruses. 2019 Jul 19;11(7):662. doi: 10.3390/v11070662 (PMC6669521; doi:10.3390/v11070662)
Supplement: Supplementary file 1 [file viruses-11-00662-s001.pdf]

SUPPLEMENTARY MATERIALS

Table S1. Antibody marker combinations used for PBMC phenotype analysis through flow cytometry.

Antibody-Marker Combination	Panel	Dilution	Species specificity	Source
CD4-FITC	A	1:200	Cat	SouthernBiotech
CD8-PE	A	1:200	Cat	SouthernBiotech
CD25-PE/Cy7[a]	A	1:20	Cat	Dr. Gregg Dean[c]
CD134-647	A	1:20	Cat	AbD Serotec
Fas-APC/Cy7[b]	A, B	1:200	Cat	R&D Systems
CD56-APC	B	1:20	Human	BioLegend
CD14-PE	B	1:40	Human	Caltag Medsystems
CD21-PE/Cy7	B	1:20	Human	BD Pharmingen
MHCII-FITC	B	1:20	Human	BD Pharmingen

a = antibody conjugated to fluorophore with an Abcam conjugation kit (ab102903), b = antibody conjugated to fluorophore with an Abcam conjugation kit (ab102859), c = Colorado State University.

Table S2. FFV prevalence and chi-square analysis data for CKD studies. Five cats were omitted from sex-specific analyses due to lack of sex data.

**Prevalence Data**									**Chi-square (X^2) Data**		
		**FFV+**		**FFV-**		**Total**	**Subgroup**	**Prevalence**	**Variable**	**X^2 Statistic**	**P-value**
**All**	**CKD+**	59	47%	28	22%	87	**All**	**67%**	**Sex vs. FFV**	0.000	1.000
	**CKD-**	25	20%	13	10%	38	CKD+FFV+	68%	(Sex ∣ CKD+) vs. FFV	0.142	0.707
	**Total**	84	67%	41	33%	125	CKD-FFV+	66%	(Sex ∣ CKD-) vs. FFV	0.468	0.494
**Male**		**FFV+**		**FFV-**		**Total**	**Subgroup**	**Prevalence**			
	**CKD+**	27	48%	10	18%	37	**M FFV+**	**68%**	**Sex vs. CKD**	0.091	0.763
	**CKD-**	11	20%	8	14%	19	M CKD+FFV+	73%	(Sex ∣ FFV+) vs. CKD	0.002	0.967
	**Total**	38	68%	18	32%	56	M CKD-FFV+	58%	(Sex ∣ FFV-) vs. CKD	0.845	0.358
**Female**		**FFV+**		**FFV-**		**Total**	**Subgroup**	**Prevalence**			
	**CKD+**	30	47%	15	23%	45	**F FFV+**	**69%**	**FFV vs. CKD**	0.039	0.844
	**CKD-**	14	22%	5	8%	19	F CKD+FFV+	67%	(FFV ∣ M) vs CKD	0.709	0.400
	**Total**	44	69%	20	31%	64	F CKD-FFV+	74%	(FFV ∣ F) vs. CKD	0.067	0.796
